# Supplementary material for: Atlasing white matter and grey matter joint contributions to resting-state networks in the human brain
Source: Commun Biol. 2023 Jul 14;6:726. doi: 10.1038/s42003-023-05107-3 (PMC10349117; doi:10.1038/s42003-023-05107-3)
Supplement: Supplementary file 3 — Description of Additional Supplementary Files [file 42003_2023_5107_MOESM3_ESM.pdf]

Description of Additional Supplementary Files

File name: Supplementary Data 1

Description: The source data behind Figures 5b and 6.
