# Supplementary material for: Atlasing white matter and grey matter joint contributions to resting-state networks in the human brain
Source: Commun Biol. 2023 Jul 14;6:726. doi: 10.1038/s42003-023-05107-3 (PMC10349117; doi:10.1038/s42003-023-05107-3)
Supplement: Supplementary file 5 — Reporting Summary [file 42003_2023_5107_MOESM5_ESM.pdf]

nature portfolio

Corresponding author(s): Marc Joliot, Victor Nozais

Last updated by author(s): 29/6/23

Reporting Summary

Nature Portfolio wishes to improve the reproducibility of the work that we publish. This form provides structure for consistency and transparency in reporting. For further information on Nature Portfolio policies, see our Editorial Policies and the Editorial Policy Checklist.

Statistics

For all statistical analyses, confirm that the following items are present in the figure legend, table legend, main text, or Methods section.

n/a	Confirmed	
☐	☒	The exact sample size (*n*) for each experimental group/condition, given as a discrete number and unit of measurement
☐	☒	A statement on whether measurements were taken from distinct samples or whether the same sample was measured repeatedly
☐	☒	The statistical test(s) used AND whether they are one- or two-sided<br>*Only common tests should be described solely by name; describe more complex techniques in the Methods section.*
☒	☐	A description of all covariates tested
☐	☒	A description of any assumptions or corrections, such as tests of normality and adjustment for multiple comparisons
☐	☒	A full description of the statistical parameters including central tendency (e.g. means) or other basic estimates (e.g. regression coefficient) AND variation (e.g. standard deviation) or associated estimates of uncertainty (e.g. confidence intervals)
☐	☒	For null hypothesis testing, the test statistic (e.g. *F*, *t*, *r*) with confidence intervals, effect sizes, degrees of freedom and *P* value noted<br>*Give P values as exact values whenever suitable.*
☒	☐	For Bayesian analysis, information on the choice of priors and Markov chain Monte Carlo settings
☒	☐	For hierarchical and complex designs, identification of the appropriate level for tests and full reporting of outcomes
☐	☒	Estimates of effect sizes (e.g. Cohen's *d*, Pearson's *r*), indicating how they were calculated

Our web collection on statistics for biologists contains articles on many of the points above.

Software and code

Policy information about availability of computer code

Data collection	Data from the publicly available Human Connectome Project database.
Data analysis	BCBtoolkit 4.1; Functionnectome 1.1.7; open software FSL 6.0, Python 3, Surf Ice, MICCA.<br>Code available with the manuscript:<br>https://github.com/NotaCS/Functionnectome<br>https://www.gin.cnrs.fr/fr/outils/micca/

For manuscripts utilizing custom algorithms or software that are central to the research but not yet described in published literature, software must be made available to editors and reviewers. We strongly encourage code deposition in a community repository (e.g. GitHub). See the Nature Portfolio guidelines for submitting code & software for further information.

Data

Policy information about availability of data

All manuscripts must include a data availability statement. This statement should provide the following information, where applicable:

- Accession codes, unique identifiers, or web links for publicly available datasets
- A description of any restrictions on data availability
- For clinical datasets or third party data, please ensure that the statement adheres to our policy

All WhiteRest unthresholded maps can be freely accessed on NeuroVault:

Grey matter maps: https://neurovault.org/collections/11937/
White matter maps: https://neurovault.org/collections/11895/
All the raw anatomical and functional data are available on the HCP website:
https://www.humanconnectome.org

Human research participants

Policy information about studies involving human research participants and Sex and Gender in Research.

Reporting on sex and gender	Sex- and gender-based analyses were not relevant to the studies objectives
Population characteristics	HCP dataset (resting-state fMRI) : 50% male/females; age range 22-35 years<br>Stroke dataset
Recruitment	HCP participants: See the HCP recruitement procedure<br>Stroke dataset: Stroke patients were recruited from the stroke service at Barnes-Jewish Hospital
Ethics oversight	Human Connectome Project<br>WashU Institutional Review Board

Note that full information on the approval of the study protocol must also be provided in the manuscript.

Field-specific reporting

Please select the one below that is the best fit for your research. If you are not sure, read the appropriate sections before making your selection.

☒ Life sciences ☐ Behavioural & social sciences ☐ Ecological, evolutionary & environmental sciences

For a reference copy of the document with all sections, see nature.com/documents/nr-reporting-summary-flat.pdf

Life sciences study design

All studies must disclose on these points even when the disclosure is negative.

Sample size	The n=150 sample size was chosen as arbitrarily large and should be sufficient not to require a detailed explanation.
Data exclusions	No data was excluded
Replication	No specific attempt at replication were done as the method is designed to create group resting-state networks (RSN) from individual RSNs reproducible across participants.
Randomization	N/A
Blinding	N/A

Reporting for specific materials, systems and methods

We require information from authors about some types of materials, experimental systems and methods used in many studies. Here, indicate whether each material, system or method listed is relevant to your study. If you are not sure if a list item applies to your research, read the appropriate section before selecting a response.

Materials & experimental systems

n/a	Involved in the study	
☒	☐	Antibodies
☒	☐	Eukaryotic cell lines
☒	☐	Palaeontology and archaeology
☒	☐	Animals and other organisms
☐	☒	Clinical data
☒	☐	Dual use research of concern

Methods

n/a	Involved in the study	
☒	☐	ChIP-seq
☒	☐	Flow cytometry
☐	☒	MRI-based neuroimaging

Clinical data

Policy information about clinical studies

All manuscripts should comply with the ICMJE guidelines for publication of clinical research and a completed CONSORT checklist must be included with all submissions.

Clinical trial registration	N/A
Study protocol	See https://doi.org/10.1016/j.neuron.2015.02.027
Data collection	See https://doi.org/10.1016/j.neuron.2015.02.027
Outcomes	N/A

Magnetic resonance imaging

Experimental design

Design type	Resting-state
Design specifications	15 min per resting-state session
Behavioral performance measures	N/A

Acquisition

Imaging type(s)	Functional
Field strength	3T
Sequence & imaging parameters	Gradient-echo EPI acquisitions using a 32-channel head coil with a multi-band acceleration factor of 8. The parameters were set with: TR=720ms, TE=33.1ms, 72 slices, 2.0mm isotropic voxels, in-plane FOV=208×180mm, flip angle=52°, BW=2290Hz/Px. Each resting-state acquisition consisted of 1200 frames (14min and 24sec), and was repeated twice using a right-to-left and a left-to-right phase encoding.
Area of acquisition	Whole brain

Diffusion MRI ☐ Used ☒ Not used

Preprocessing

Preprocessing software	The functional data used in this study is the preprocessed data from the Human connectome database. It has been preprocessed using the "Minimal Preprocessing Pipelines" of the Human Connectome Project. It uses FreeSurfer et FSL tools. More detail are available of the HCP website (https://www.humanconnectome.org/software/hcp-mr-pipelines) and the associated publication (Glasser et al. 2013, http://doi.org/10.1016/j.neuroimage.2013.04.127)
Normalization	As per the HCP pipelines (FLIRT, FNIRT)
Normalization template	MNI152 2mm isotropic
Noise and artifact removal	As per the HCP pipelines
Volume censoring	As per the HCP pipelines

Statistical modeling & inference

Model type and settings	N/A
Effect(s) tested	N/A

Specify type of analysis: ☒ Whole brain ☐ ROI-based ☐ Both

Statistic type for inference (See Eklund et al. 2016)	Voxel-wise z-stat
Correction	No correction

Models & analysis

n/a	Involved in the study
☒	☐ Functional and/or effective connectivity
☒	☐ Graph analysis
☒	☐ Multivariate modeling or predictive analysis
